# Supplementary material for: π-π-Stacked Poly(ε-caprolactone)-b-poly(ethylene glycol) Micelles Loaded with a Photosensitizer for Photodynamic Therapy
Source: Pharmaceutics. 2020 Apr 9;12(4):338. doi: 10.3390/pharmaceutics12040338 (PMC7238042; doi:10.3390/pharmaceutics12040338)
Supplement: Supplementary file 1 [file pharmaceutics-12-00338-s001.pdf]

pharmaceutics

Supplementary Materials: π-π Stacked Poly(ε-caprolactone)-*b*-poly(ethylene glycol) Micelles Loaded With Photosensitizer for Photodynamic Therapy

Yanna Liu, Marcel H.A.M. Fens, Bo Lou, Nicky C.H. van Kronenburg, Roel F.M. Maas-Bakker, Robbert J. Kok, Sabrina Oliveira, Wim E. Hennink and Cornelus F. van Nostrum *

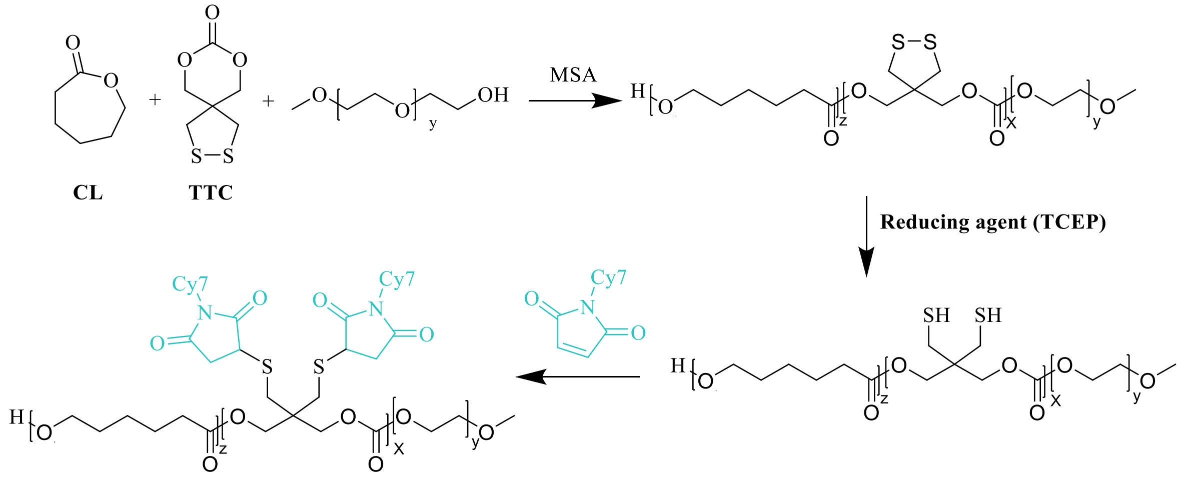

Scheme S1. Synthesis and Cy7 labeling of P(CL_{18}-$TTC_{7.5}$)-PEG [1].

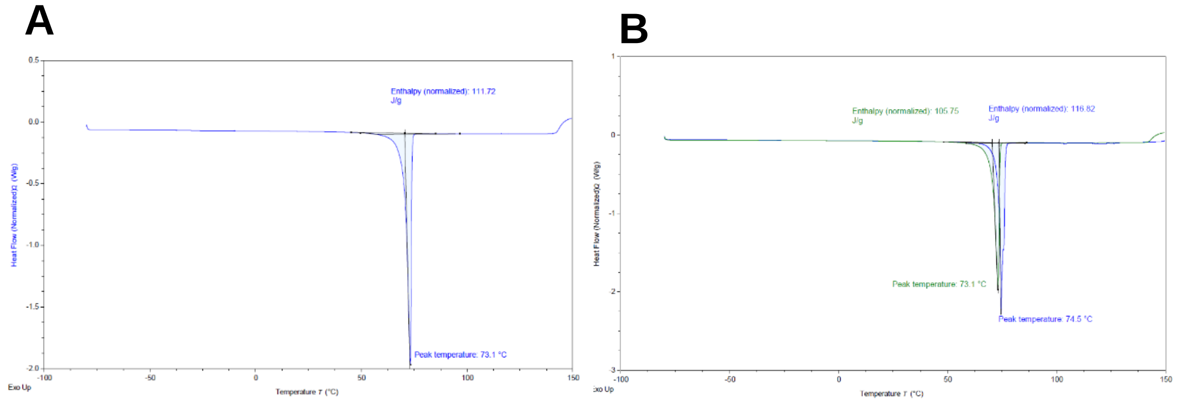

Figure S1. Thermograms of benzyl 2,2-bis(hydroxymethyl)propionate (A) and benzyl 5-methyl-2-oxo-1,3-dioxane-5-carboxylate (before (green line) and after (blue line) recrystallization) (B), recorded by DSC.

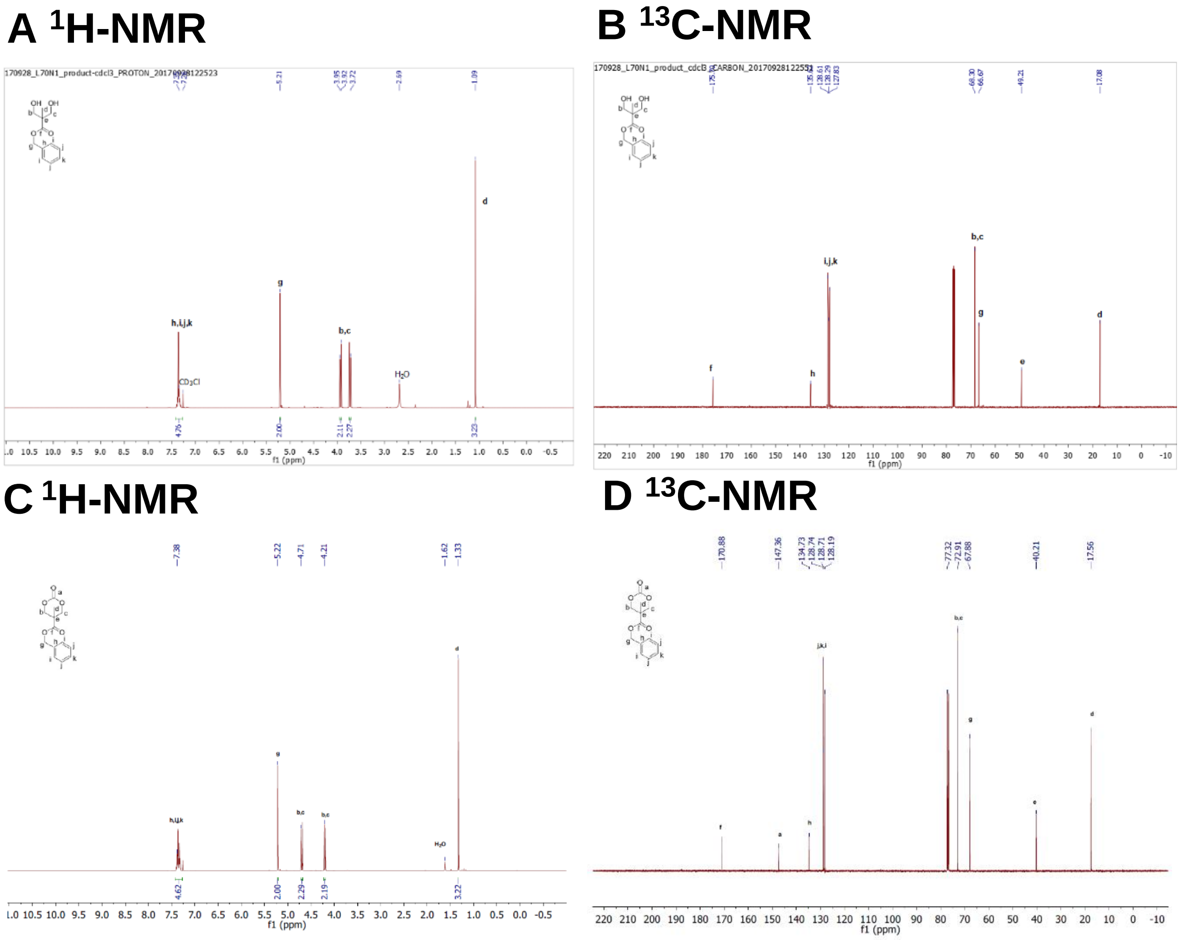

Figure S2. ^1H/^{13}C NMR spectra of the benzyl 2,2-bis(methylol)propionate intermediate (A, B) and benzyl 5-methyl-2-oxo-1,3-dioxane-5-carboxylate (*i.e.*, TMC-Bz) (C, D).

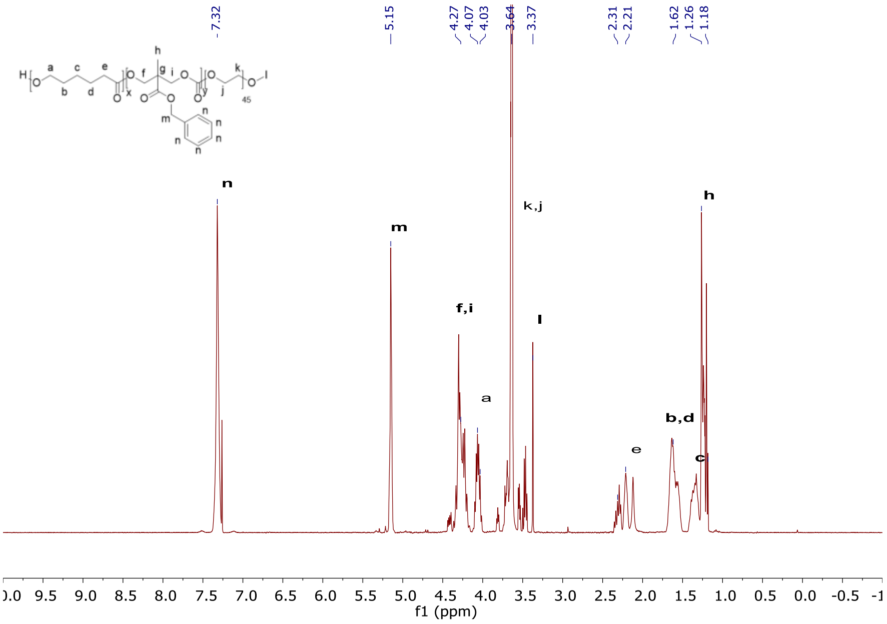

Figure S3. ^1H NMR spectrum of P(CL-TMC-Bz)-PEG. ^1H-NMR (600 MHz, $CDCl_3$): δ 7.34 (m, CH_2***C_6H_5***), 5.15 (s, ***CH_2***C_6H_5), 4.30–4.05 (m, COO***CH_2***C***CH_2***OCO, $COCH_2CH_2CH_2CH_2$***CH_2***O), 3.64 (m, PEG protons), 3.38 (s, 3H, ***CH_3***O), 2.30 (m, CH_2CH_2***CH_2***COO), 1.62 (m, CH_2***CH_2***CH_2***CH_2***CH_2), 1.34 (m, CH_2CH_2***CH_2***CH_2CH_2), 1.27-1.21 (m, OCH_2C***CH_3***).

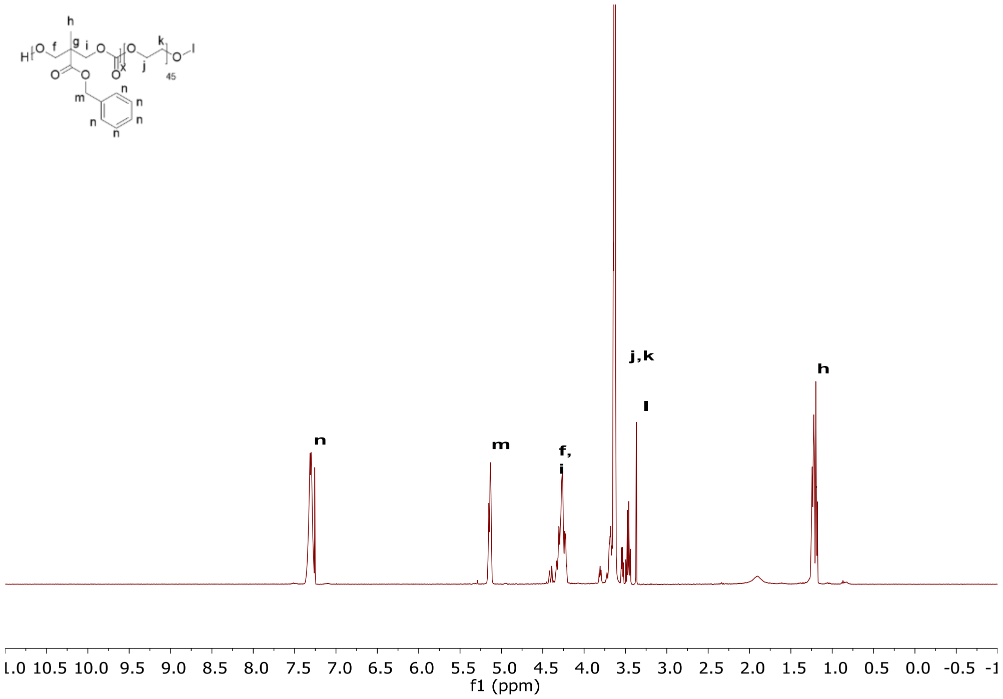

Figure S4. ^{1}H NMR spectrum of P(TMC-Bz)-PEG. ^{1}H-NMR (600 MHz, $CDCl_3$,): δ 7.34 (m, $CH_2\underline{C_6H_5}$), 5.15 (s, $\underline{CH_2}C_6H_5$), 4.30–4.05 (m, $COO\underline{CH_2}C\underline{CH_2}OCO$, $COCH_2CH_2CH_2CH_2\underline{CH_2}O$), 3.64 (m, PEG protons), 3.38 (s, 3H, $\underline{CH_3}O$), 1.28 (m, $OCH_2C\underline{CH_3}$).

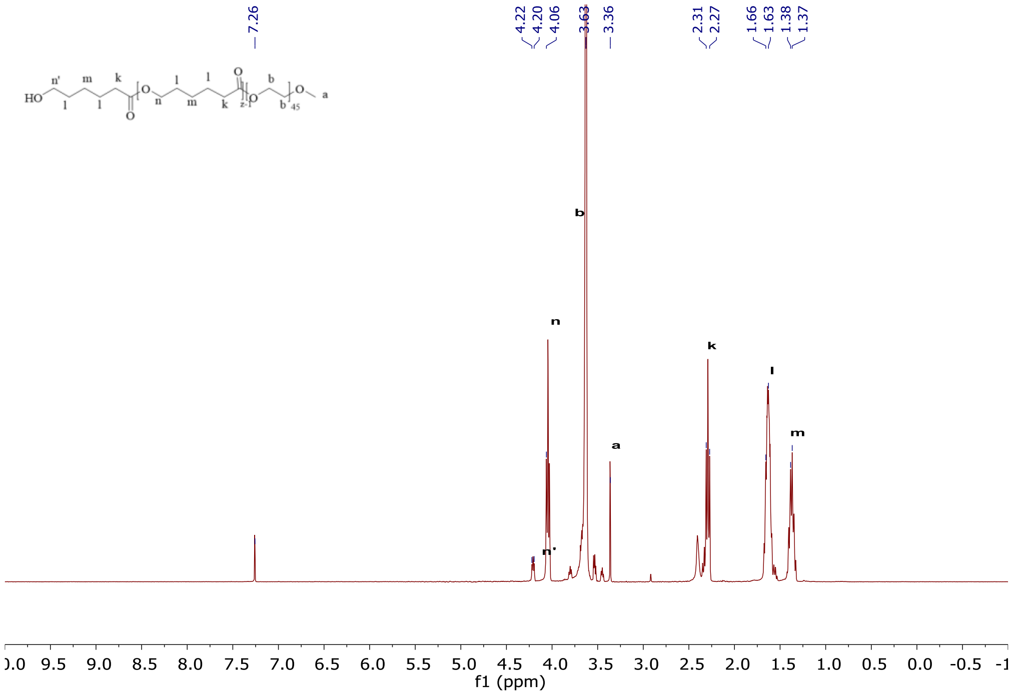

Figure S5. ^{1}H NMR spectrum of PCL-PEG. ^{1}H-NMR (600 MHz, $CDCl_3$,): δ 4.29–4.00 (m, $COO\underline{CH_2}$, $CH_2\underline{CH_2}OH$), 3.64 (m, PEG protons), 3.37 (s, $\underline{CH_3}O$), 2.29 (m, $CH_2CH_2\underline{CH_2}COO$), 1.66 (m, $CH_2\underline{CH_2}CH_2\underline{CH_2}CH_2$), 1.38 (m, $CH_2CH_2\underline{CH_2}CH_2CH_2$).

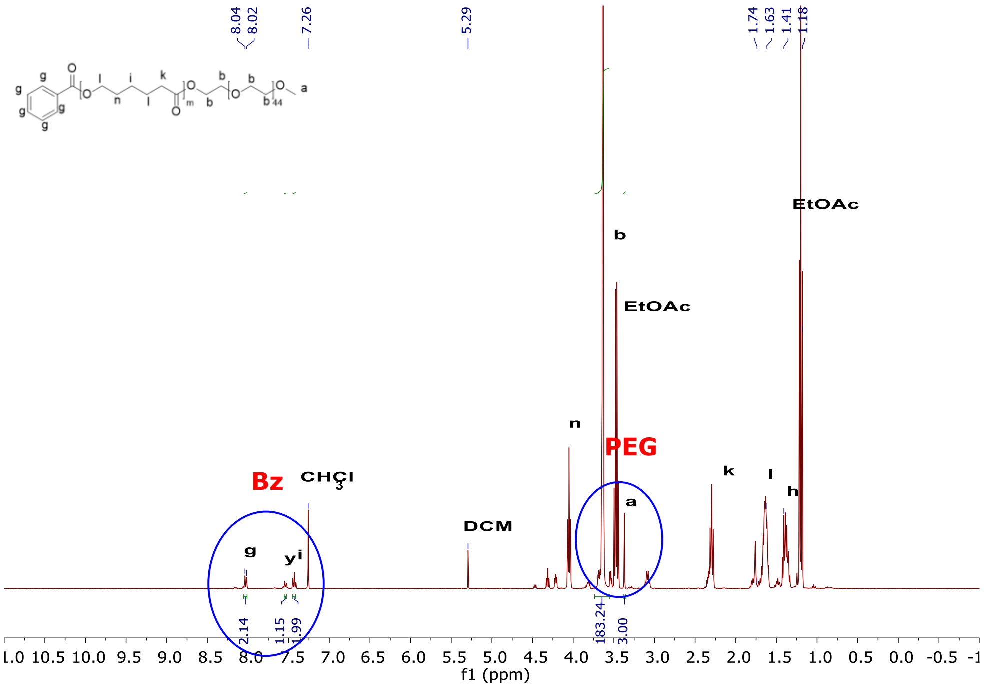

Figure S6. ^{1}H NMR spectrum of Bz-PCL-PEG. ^{1}H-NMR (600 MHz, $CDCl_3$): δ 8.0 (d, 2H, aromatic CH), 7.53 (t, 1H, aromatic CH), 7.41 (t, 2H, aromatic CH), 4.29 (t, 2H, $C_6H_5COO\underline{\mathit{CH_2}}$), 4.20–4.00 (m, $COO\underline{\mathit{CH_2}}$, $CH_2\underline{\mathit{CH_2}}OH$), 3.64 (m, PEG protons), 3.37 (s, $\underline{\mathit{CH_3}}O$), 2.29 (m, $CH_2CH_2\underline{\mathit{CH_2}}COO$), 1.66 (m, $CH_2\underline{\mathit{CH_2}}CH_2\underline{\mathit{CH_2}}CH_2$), 1.38 (m, $CH_2CH_2\underline{\mathit{CH_2}}CH_2CH_2$).

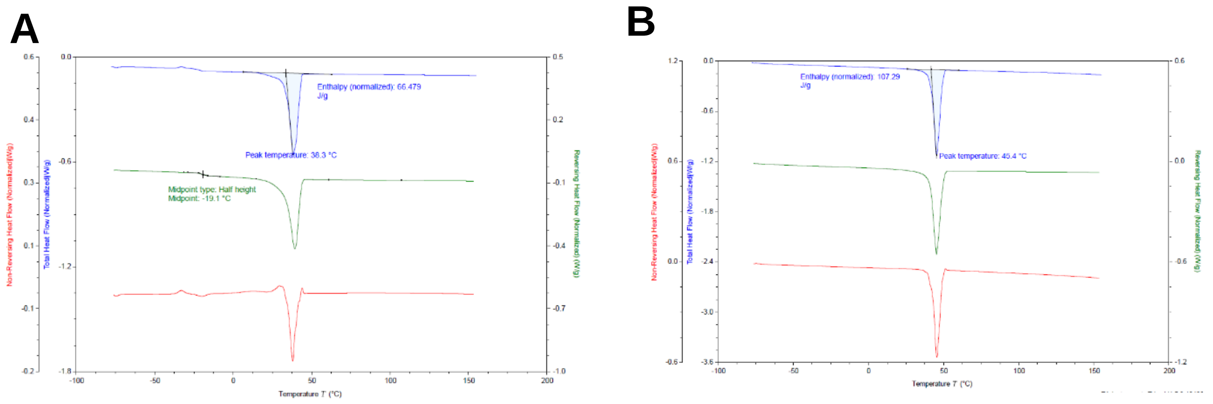

Figure S7. Thermograms of P($CL_{9.1}$-TMC-$Bz_{7.7}$)-PEG (Entry 7, table 1) and $PCL_{17.6}$-PEG (Entry 5, table 1), recorded by DSC.

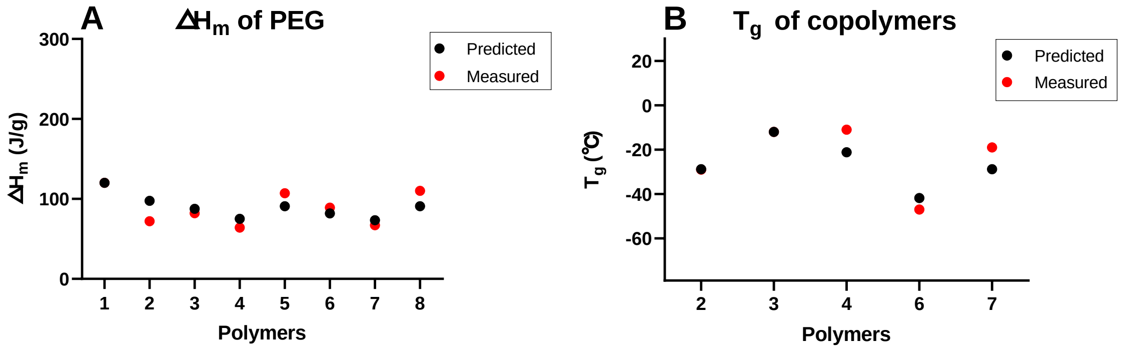

Figure S8. (A) Measured $\triangle H_m$'s of PEG in the synthesized block copolymers (red dots), corrected for the weight fraction of PEG of the block copolymer. The predicted $\triangle H_m$'s (black dots) were obtained by using mPEG-OH (measured $\triangle H_m$ of 182 J/g and the weight fraction of PEG in the block copolymers) as the reference. (B) Measured T_g's of the synthesized P(CL-TMC-Bz)-PEG copolymers (red dots) with random CL and TMC-Bz sequence. Predicted T_g's (black dots) were calculated based on FOX equation in which T_g of −60 °C for high molecular weight PCL_{80} [2] and T_g of −12 °C for P(TMC-Bz) (obtained from P(TMC-$Bz_{8.6}$)-PEG, Entry 3, table 1) were used as the reference for the prediction. The numbers of the polymers on the x-axis correspond to the same entries in table 1.

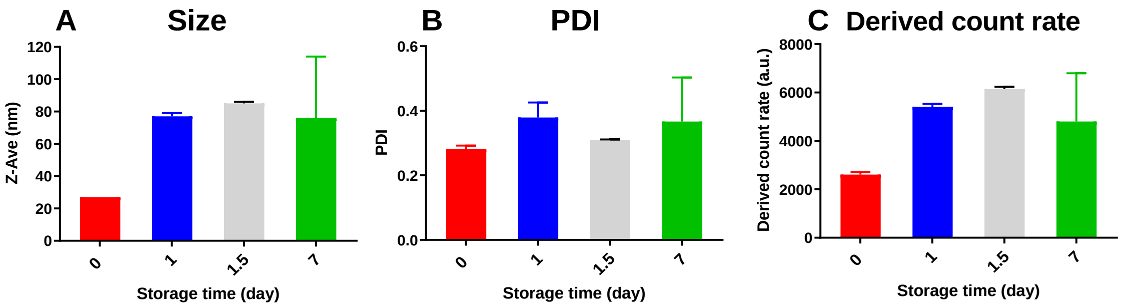

Figure S9. Size (A), PDI (B) and derived count rate (C) of PCL_9-PEG micelles in PBS after storage at room temperature over a period of 7 days.

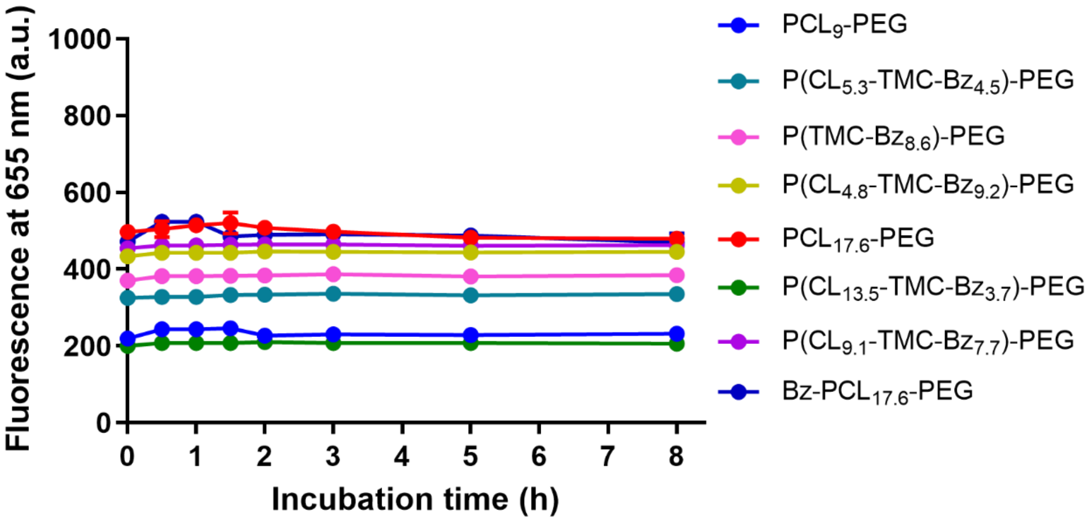

Figure S10. Fluorescence intensity (λ_{ex} 420 nm, λ_{em} 655 nm) as a function of time at 37 °C in PBS; micelles of 10 mg/mL with 5 wt% loading amounts were prepared and diluted 10× in PBS, to obtain the final mTHPC concentration of 40 μg/mL.

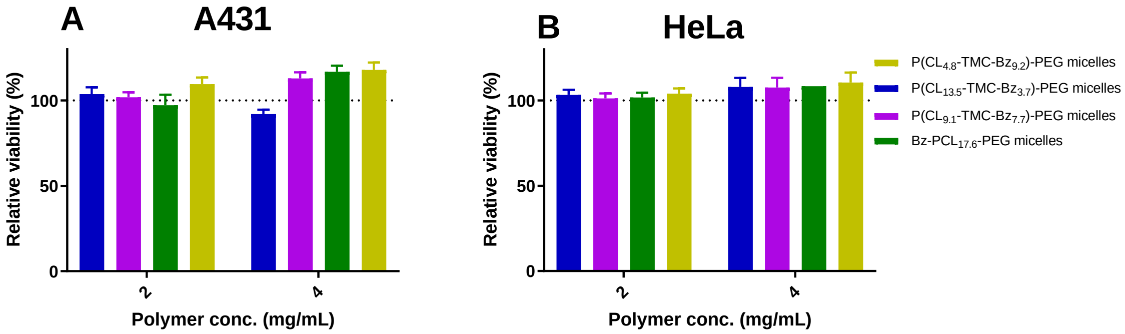

Figure S11. Cytotoxicity by MTS assay of different empty micelles composed of 2 and 4 mg/mL polymer on A431 and HeLa cells after 24 h incubation (n = 3).

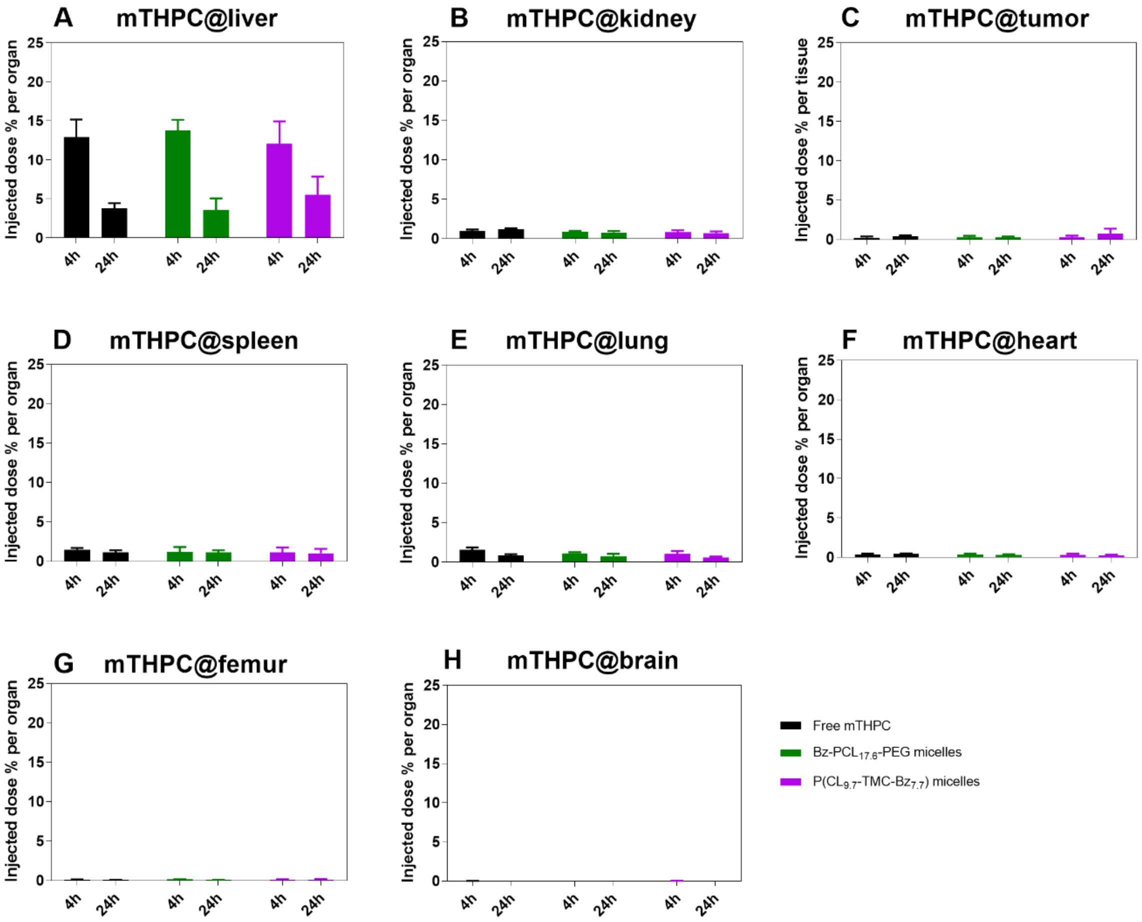

Figure S12. Biodistribution of free mTHPC and mTHPC loaded in micelles in tumor and main organs of mice after 4 and 24 h administration of the formulations at a mTHPC dose of 0.3 mg/kg. Data are indicated as the percentage of the injected mTHPC (%ID) present per organ/tumor (n = 3-5).

Reference

1. Liu, Y.; Scrivano, L.; Peterson, J.D.; Fens, M.H.A.M.; Hernández, I.B.; Mesquita, B.; Toraño, J.S.; Hennink, W.E.; Nostrum, C.F.; Oliveira, S. EGFR targeted nanobody functionalized polymeric micelles loaded with mTHPC for selective photodynamic therapy. *Mol. Pharmaceutics* **2020**, *17*, 1276–1292.
2. Couffin, A.; Delcroix, D.; Martín-Vaca, B.; Bourissou, D.; Navarro, C. Mild and efficient preparation of block and gradient copolymers by methanesulfonic acid catalyzed ring-opening polymerization of caprolactone and trimethylene carbonate. Macromolecules **2013**, 46, 4354-4360.
